# Supplementary material for: Causal Relationship between Aging and Anorexia Nervosa: A White-Matter-Microstructure-Mediated Mendelian Randomization Analysis
Source: Biomedicines. 2024 Aug 16;12(8):1874. doi: 10.3390/biomedicines12081874 (PMC11351342; doi:10.3390/biomedicines12081874)
Supplement: Supplementary file 1 [file biomedicines-12-01874-s001.zip › Supplementary Figure/Figure S4/Singe SNP plot of MR analysis for TL to ubm-b-1800.pdf]

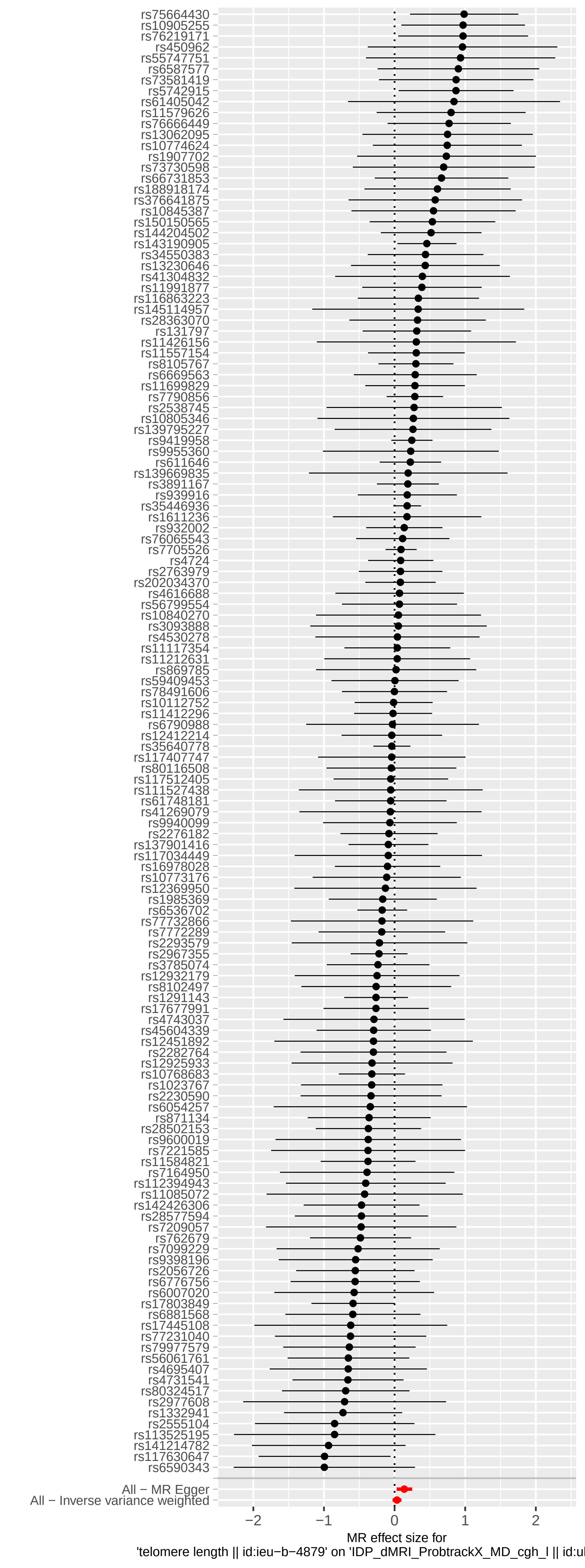
rs75664430
rs10905255
rs76219171
rs450962
rs55747751
rs6587577
rs73581419
rs5742915
rs61405042
rs11579626
rs76666449
rs13062095
rs10774624
rs1907702
rs73730598
rs66731853
rs188918174
rs376641875
rs10845387
rs150150565
rs144204502
rs143190905
rs34550383
rs13230646
rs41304832
rs11991877
rs116863223
rs145114957
rs28363070
rs131797
rs11426156
rs11557154
rs8105767
rs6669563
rs11699829
rs7790856
rs2538745
rs10805346
rs139795227
rs9419958
rs9955360
rs611646
rs139669835
rs3891167
rs939916
rs35446936
rs1611236
rs932002
rs76065543
rs7705526
rs4724
rs2763979
rs202034370
rs4616688
rs56799554
rs10840270
rs3093888
rs4530278
rs11117354
rs11212631
rs869785
rs59409453
rs78491606
rs10112752
rs11412296
rs6790988
rs12412214
rs35640778
rs117407747
rs80116508
rs117512405
rs111527438
rs61748181
rs41269079
rs9940099
rs2276182
rs137901416
rs117034449
rs16978028
rs10773176
rs12369950
rs1985369
rs6536702
rs77732866
rs7772289
rs2293579
rs2967355
rs3785074
rs12932179
rs8102497
rs1291143
rs17677991
rs4743037
rs45604339
rs12451892
rs2282764
rs12925933
rs10768683
rs1023767
rs2230590
rs6054257
rs871134
rs28502153
rs9600019
rs7221585
rs11584821
rs7164950
rs112394943
rs11085072
rs142426306
rs28577594
rs7209057
rs762679
rs7099229
rs9398196
rs2056726
rs6776756
rs6007020
rs17803849
rs6881568
rs17445108
rs77231040
rs79977579
rs56061761
rs4695407
rs4731541
rs80324517
rs2977608
rs1332941
rs2555104
rs113525195
rs141214782
rs117630647
rs6590343
All − MR Egger
All − Inverse variance weighted
−2
−1
0
1
2
MR effect size for
'telomere length || id:ieu−b−4879' on 'IDP_dMRI_ProbtrackX_MD_cgh_l || id:ul
